# Early career interview: Nathan Bahr

**DOI:** 10.2144/fsoa-2019-0144

**Published:** 2020-01-27

**Authors:** Nathan Bahr

**Affiliations:** 1 University of Kansas Division of Infectious Diseases, Department of Medicine 3901 Rainbow Boulevard Mail Stop 1028, Kansas City, KS 66160, USA

**Keywords:** cryptococcal meningitis and histoplasmosis, global health, tuberculosis meningitis

## Abstract

Nathan Bahr is an Assistant Professor in infectious disease research at the University of Kansas (USA). He works in global health, tuberculosis meningitis, cryptococcal meningitis and histoplasmosis. He was one of three finalists of the 2019 Future Science Future Star Award. Here, he tells us about his career to-date.

## Please tell us about yourself

I have been fortunate to become involved with an innovative group studying central nervous system (CNS) infections among persons with HIV. After completing my medical school at Loyola University, Chicago (IL, USA) combined with an MA in bioethics and health policy, I went to the University of Minnesota (USA) where I completed internal medicine residency followed by a chief resident year. I then began ID fellowship at the University of Minnesota, my first year being spent in Uganda via a NIH Fogarty fellowship. After my second year, I transferred to the University of Kansas for my final year due to my wife's matching there for her own fellowship. I was then hired as faculty and I will complete my third year on faculty shortly. To date I have 47 publications, of which 36 were peer reviewed and 21 were either first or last author. Two of my studies have directly led to changes in WHO guidelines. My focus is primarily on improving the diagnosis of TB meningitis (TBM) and I have recently been awarded an NIH K23 award starting 1 July to study TBM and histoplasmosis.

## What made you choose a career in your field?

Infectious diseases is a field that requires detective work and really a unique frame of mind. CNS infections often have high mortality – in my research I am looking at conditions that are often understudied and so there is ample space to make giant advances for patients.

## What are the main highlights of your career so far?

Receiving the K23 award, starting on 1 July 2019. The WHO guidelines reflecting my protocol for electrolyte management in cryptococcal meningitis treated with amphotericin (2014) and WHO guideline reflecting my study of GeneXpert Ultra, now recommended as the first test for diagnosis of TBM (2018).

## What is the most difficult challenge you have encountered in your work & how did you overcome it?

When I spent a year in Uganda I had just become engaged. The work went great but it was hard to be away from my fiancé (now wife). That year taught me many lessons about balancing work and life. It was a big part of why we ended up moving to Kansas and that decision helped me think of research differently – it is now part of my career for which I have great passion, rather than being enjoyable, but something I was not really sure about. When we moved to Kansas, I did not have much time for research, that was a clinical year. Yet, I found time to be quite productive in a way that balanced my home life and my work. These lessons have made me efficient and more effective in all of my roles. I am a better physician, researcher, husband and now father for having pushed through that extended time in Uganda and the time where I did not have formal time for research in Kansas. I still travel to Uganda, but our team-based approach makes these short trips enjoyable – not taxing.

## What is your favorite publication so far?

I would say [1]. This study was the first study published using this test for TBM and the accompanying editorial suggested it might be a game changer for diagnosis of TBM. This is a disease in which traditional diagnostics are at best 40–50% sensitive and require weeks for a result. Our results showed at least 70% sensitivity – possibly up to 95% sensitivity in around 1–2 h. This study has impressive potential to change mortality for the better in TBM be lessening missed or delayed diagnoses.

## What are your main aims for the future?

To improve TBM diagnosis through adjunctive immunodiagnostic techniques, improve histoplasmosis diagnosis through immunodiagnostic techniques, gain skills in biostatistics, trial design and diagnostic test development to set myself up for a career as an independent investigator studying diagnostic tests for CNS infections.

## Where do you hope to see yourself in 5 years?

With a newly awarded R01 from the NIH to continue to study and improve diagnosis and treatment of TBM.

## References

[1] Bahr NC, Nuwagira E, Evans EE Diagnostic accuracy of Xpert MTB/RIF Ultra for tuberculous meningitis in HIV-infected adults: a prospective cohort study. Lancet Infect. Dis. 18(1), 68–75 (2018). 28919338 10.1016/S1473-3099(17)30474-7 PMC5739874

